# Emotional Comparison Between Semantic Dementia and Alzheimer's Disease

**DOI:** 10.3389/fpsyt.2021.680332

**Published:** 2021-05-13

**Authors:** Ping Wang, Qianhua Zhao, Yan Zhou, Zhen Hong, Qihao Guo, Jianren Liu

**Affiliations:** ^1^Department of Neurology, Shanghai Ninth People's Hospital, Shanghai JiaoTong University School of Medicine, Shanghai, China; ^2^Department of Neurology, Huashan Hospital, Fudan University, Shanghai, China; ^3^Department of Geriatrics, Shanghai Jiaotong University Affiliated Sixth People's Hospital, Shanghai, China

**Keywords:** anxiety, depression, apathy, empathy, alzhaimer's disease, semantic dementia

## Abstract

**Background:** Previous studies have suggested that Alzheimer's disease (AD) and semantic dementia (SD) are both associated with emotional processing impairment. However, the degree and type of emotional symptoms between the two types of dementia have not been previously compared.

**Method:** We used the Apathy Evaluation Scale (AES), the Toronto Empathy Questionnaire (TEQ), the Geriatric Depression Scale (GDS) and the Self-rating Anxiety Scale (SAS) to examine apathy, empathy, depression and anxiety, respectively.

**Results:** Between mild AD and mild SD, moderate-to-severe AD and moderate-to-severe SD, the total scores of TEQ are significantly different, but the total scores of GDS, SAS and AES have no significant differences. In addition, normal individuals, AD and SD patients got the similar scores in SAS and GDS.

**Conclusions:** Empathy emotion in SD patients is more severe than that in AD patients. However, apathy, depression and anxiety emotion is similar between the two groups.

## Introduction

Semantic dementia (SD; also known as semantic variant primary progressive aphasia) is one of the clinical variants of frontotemporal dementia (FTD), in which naming problems and single-word comprehension are both severely impaired due to asymmetric atrophy (generally left greater than right) in the anterior temporal lobes (1). In contrast, Alzheimer disease (AD) is clinically characterized by prominent episodic memory disturbance affecting learning and retrieval of newly learnt information, accompanied by deficits in at least one other cognitive domain (ie, visuospatial, language, and executive functions) (2).

Emotional changes such as apathy, empathy, depression and anxiety are the most common symptoms in all-cause dementia and are strongly associated with increased caregiver burden and lower quality of life in people with dementia, but the relationship has yet to be determined. Some studies suggest that emotional changes are accompanying symptoms of dementia, while others consider there is a link between emotional symptoms and dementia. One possible explanation is that emotional illness causes dementia, while another possibility is that dementia triggers a relapse of emotional symptoms (3). In order to determine the relationship, it is first necessary to know the differences of emotional symptoms between normal individuals and patients of dementia. The next step is to determine whether the emotional changes are different in different types and stages of dementia. This study focuses on the four emotional symptoms (apathy, empathy, depression and anxiety) in AD, SD and normal individuals, in order to provide better care advice to different dementia patients.

Depressive symptoms are very common in dementia. In AD, the prevalence of depression varies from 6 to 42% (4). In SD, the reported prevalence is 44–78%. The proportion seems to be relatively similar across dementia stages and is higher in patients with vascular dementia (VaD) and FTD than in AD (3, 5).

Apathy is a symptom defined as lack of motivation not attributable to diminished level of consciousness, cognitive impairment, or emotional distress related to daily function (6). It is one of the most prevalent and disabling non-cognitive symptoms of dementia, affecting up to 90% of individuals over the disease course (7). Sixty percent of AD and 84% of behavioral variant frontotemporal dementia (bvFTD) patients had some degree of apathy, and bvFTD patients had more severe and more frequent symptoms than AD (8). Some studies found that the rate of apathy in SD is lower than those seen in disorders involving cortical dysfunction, such as AD and Traumatic Brain Injury (9, 10).

There has been a growing body of research supporting an association between anxiety and dementia. It is more common in people who are cognitively impaired than in those who are not (11). A meta-analysis shows that the overall prevalence of anxiety in people with dementia was 14% (3). In AD, the pooled prevalence of anxiety is reportedly 39% (12). While in SD, the anxiety symptoms are ranging from 41 to 56% (13). Considering the severity of dementia, anxiety symptoms are generally equally prevalent at mild and moderate levels of severity but decrease at the severe and profoundly demented stage (14).

Empathy is the ability to share the emotions and sensations of others. It is often characterized as the ability to “put oneself into another's shoes,” or in some way experience another person's emotions within oneself. This is crucial for a higher social functioning, and when impaired, difficulty with social conduct and relationship turmoil is observed. The loss of empathy is an early symptom reported by carers of patients with FTD (15–17). Mendez et al. analyzed empathy in patients with bvFTD and indicated that these patients had decreased empathic behavior with or without emotional blunting (18).

Most studies just reported one or two of these symptoms in AD or SD. Our main objective is to simultaneously describe and compare the above four emotional symptoms of patients presenting with different stages of AD and SD. This study may contribute to a better understanding of the emotional symptoms of these patients, thus providing suggestions to caregivers in taking care of them.

## Materials and Methods

### Participants

Patients with SD or AD and cognitively normal controls (NC) were recruited from the Memory Clinic, Huashan Hospital, during Apr 2012-Feb 2018. In the present study, 165 subjects (105 men, 60 women) were enrolled including 83 AD patients, 43 SD patients and 39 NC. All of the 126 demented patients had finished the laboratory tests and cranial CT/MRI scan and were found to have no clinically significant abnormalities in vitamin B12, folic acid, thyroid function (free triiodothyronine-FT3, free tetraiodothyronine-FT4, thyroid stimulating hormone-TSH), rapid plasma regain (RPR), or treponema pallidum particle agglutination (TPPA).

AD was diagnosed as probable AD according to the National Institute of Neurological and Communicative Disorders and Stroke-Alzheimer's Disease and Related Disorders Association (NINCDS-ADRDA/NINCDS-AIREN) criteria. SD was diagnosed according to the guidelines proposed in 2011, in which anomia and single-word comprehension deficits are the core features (19). The severity of AD and SD was judged according to Clinical Dementia Rating (CDR) Scale (20) and FTLD-modified Clinical Dementia Rating (FTLD-modified CDR) Scale (21). CDR = 1, 2, or 3 indicated mild, moderate, or severe dementia.

### Emotional Scales

The emotional scales were administered by a trained rater who was blind to diagnosis. To determine the general cognitive function, all study subjects completed MMSE(Mini-Mental state examination) (22), MES (memory and executive screening scale) (23), CFT(complex figure test) (24), AVLT(auditory verbal learning test) (25), AFT(animal fluency test) (26), BNT(Boston naming test) (27), SDMT (symbol digit modalities test) and reading (26).

#### Geriatric Depression Scale

The 15-item short form developed by Sheikh and Yesavage in 1986 (28) was used for the present study. Responses were coded 1 = yes, has symptom; vs. 0 = no, symptom not present. Items were summed and higher scores indicated a greater degree of depressive symptoms.

#### Self-Rating Anxiety Scale

The SAS (29) is a 20-item measure developed to assess the frequency of anxiety symptoms based on diagnostic conceptualizations. It consists primarily of somatic symptoms. The respondent indicates how often he or she has experienced each symptom on a 4-point Likert scale consisting of “one or a little of the time” (coded as 1), “some of the time” (coded as 2), “good part of the time” (coded as 3), and “most or all of the time” (coded as 4). Items 5, 9, 13, 16, and 19 are reversed scored and total scores on the SAS range from 0 to 80.

#### Apathy Evaluation Scale

The presence of apathy was established on the Apathy Evaluation Scale. The 18-item informant-rated scale (AES-I) used here was developed by Marin et al. (30). It rated a person's thoughts, actions, and emotions over the previous 4 weeks. A score higher than 20 points is associated with an apathic syndrome. This scale was validated in participants with Alzheimer's disease and other dementias, stroke and major depression.

#### The Toronto Empathy Questionnaire

The toronto empathy questionnaire (TEQ) developed by Spreng et al. (31) is an uni-dimensional, 16-item, five-point Likert type scale to assess the empathy levels of individuals. We used the informant-report style. There are 16 items in the questionnaire and the informants are expected to express their opinions in the questionnaire ranging from “Never” to “Always” on 5-point Likert type scale with answer of “Never” been marked as “1,” “Always” as “5.” The high scores accounts for high empathy.

Validity and reliability of the Chinese versions of these four scales have been assessed.

### Ethical Compliance

This study was approved by the ethics committee of Shanghai Huashan Hospital Fudan University. All participants signed a consent form.

### Statistical Analysis

Statistical analysis was done using the SPSS package version 13.0. Overall differences among the groups were determined by chi square tests for categorical data and analysis of variance (ANOVA) for continuous variables. To eliminate the confounding factor of education years, an analysis of covariance (ANCOVA) was performed. *Post-hoc* tests for ANOVA or ANCOVA was performed to determine whether there were significant differences between pairs of groups while accounting for multiple comparisons. Correlation was done using the Pearson bivariate correlation analysis. The level of significance for all comparisons was set at *p* < 0.05.

## Results

### Demographic Features

One hundred and sixty five subjects (105 men, 60 women) were enrolled in the study. Among these, 83 were affected by AD, and 43 were diagnosed with SD. The detailed demographic features are shown in Table 1. One-way analysis of variance and chi square tests showed that the patient groups were matched on age and gender; but moderate-to-severe SD patients got significantly less education than NC group and mild AD patients, and moderate-to-severe AD patients were less educated than NC group.

**Table 1:** Demographic features of the subjects.

	**NC (*n* = 39)**	**Mild AD (*n* = 48)**	**Moderate-to-severe AD(*n* = 35)**	**Mild SD (*n* = 20)**	**Moderate-to-severe SD (*n* = 23)**	**Statistics and *P*-value**
Age	62.9 (10.7)	62.4 (11.1)	62.8 (13.3)	62.4 (7.7)	62.6 (8.3)	*F* = 0.01, *p* = 1.00
Years of education	12.3 (3.4)^c^^,^^d^	11.4 (3.1)	9.9 (3.9)^a^^,^^d^	10.8 (2.9)	8.6 (4.1)^a^^,^^b^	*F* = 5.04, *p* = 0.00
Male (*N*%)	25 (64.1%)	35 (72.9%)	25 (71.4%)	10 (50.0%)	10 (43.5%)	χ^2^ = 8.35, *p* = 0.079

*Continuous variables compared by ANOVA with post-hoc pairwise Bonferroni tests. Gender compared by chi square*.

*The scores were presented as mean (standard deviation)*.

a *p < 0.05 compared to NC group*.

b *p < 0.05 compared to mild AD group*.

c *p < 0.05 compared to moderate-to-severe AD group*.

d *p < 0.05 compared to moderate-to-severe SD group*.

### Background Neuropsychology

The subjects underwent a background neuropsychological examination. Their performance was summarized in Table 2. Most patients with moderate-to-severe SD did not perform neuropsychological evaluation because they were not able to copy a geometric figure or to read words. Based on the existing data, the results of our analysis were as follows. The difference between mild AD and mild SD, moderate-to-severe AD and moderate-to-severe SD for the MMSE score, did not reach statistical significance. MES showed no significant difference between mild AD and mild SD. For AFT, there was no significant difference between moderate-to-severe AD and mild SD. The score of AVLT-DR was lower in AD and SD than in healthy adults. For CFT-copy, CFT-DR, BNT, SDMT and reading, significant differences existed in any two of the four groups.

**Table 2:** General neuropsychology of the five groups.

	**Total score**	**NC (*n* = 39)**	**Mild AD (*n* = 48)**	**Moderate-to-severe AD (*n* = 35)**	**Mild SD (*n* = 20)**	**Moderate-to-severe SD (*n* = 23)**	***F* (*P*)**
MMSE	30	27.6 (1.8)^b^^,^^c^^,^^d^^,^^e^	21.1 (2.5)^a^^,^^c^^,^^e^	10.7 (3.9)^a^^,^^b^^,^^d^	20.4(4.3)^a^^,^^c^^,^^e^	9.2 (2.5)^a^^,^^b^^,^^d^	213.69 (0.00)
MES	100	81.7 (8.9)^b^^,^^c^^,^^d^	48.5 (12.3)^a^^,^^c^	25.8 (13.2)^a^^,^^b^^,^^d^	51.1 (17.9)^a^^,^^c^	-	107.60 (0.00)
AVLT-DR	12	4.6 (1.6)^b^^,^^c^^,^^d^	0.4 (0.9)^a^	0.1 (0.4)^a^	0.1 (0.5)^a^	-	126.31 (0.00)
CFT-copy	36	32.5 (4.5)^b^^,^^c^^,^^d^	25.1 (10.0)^a^^,^^c^^,^^d^	19.9 (10.9)^a^^,^^b^^,^^d^	31.0 (6.4)^a^^,^^b^^,^^c^	-	11.54 (0.00)
CFT-DR	36	14.5 (5.3)^b^^,^^c^^,^^d^	4.5 (3.8)^a^^,^^c^^,^^d^	3.7 (4.4)^a^^,^^b^^,^^d^	9.3 (5.1)^a^^,^^b^^,^^c^	-	26.98 (0.00)
BNT	30	24.1 (3.2)^b^^,^^c^^,^^d^	18.1 (5.2)^a^^,^^c^^,^^d^	10.0 (5.5)^a^^,^^b^^,^^d^	7.1 (4.1)^a^^,^^b^^,^^c^	-	68.74 (0.00)
AFT	-	16.7 (4.4)^b^^,^^c^^,^^d^	11.7 (3.8)^a^^,^^c^^,^^d^	8.4(4.1)^a^^,^^b^	6.7 (3.5)^a^^,^^b^	-	29.86 (0.00)
SDMT	-	34.5 (11.4)^b^^,^^c^^,^^d^	19.9 (12.4)^a^^,^^c^^,^^d^	8.6 (8.4)^a^^,^^b^^,^^d^	27.3 (8.5)^a^^,^^b^^,^^c^	-	20.51 (0.00)
Reading	50	42.8 (4.4)^b^^,^^c^^,^^d^	35.9 (6.5)^a^^,^^c^^,^^d^	28.1 (10.6)^a^^,^^b^^,^^d^	16.5 (11.4)^a^^,^^b^^,^^c^	-	52.84 (0.00)

*These variables compared by ANCOVA with post-hoc pairwise LSD tests*.

*The scores were presented as mean (standard deviation)*.

a *p < 0.05 compared to NC group*.

b *p < 0.05 compared to mild AD group*.

c *p < 0.05 compared to moderate-to-severe AD group*.

d *p < 0.05 compared to mild SD group*.

e *p < 0.05 compared to moderate-to-severe SD group*.

*MMSE, total score of Mini-Mental state examination; MES, memory and executive screening scale; AVLT- DR, delayed recall of auditory verbal learning test; CFT-Copy, copy part of complex figure test; CFT-DR, delayed recall of complex figure test; BNT, Boston naming test; AFT, animal fluency test; SDMT, symbol digit modalities test*.

In all, AD is characterized by impaired episodic memory, while SD mainly has semantic memory disturbance. The severity of memory impairment is close between mild AD and mild SD, as well as between moderate-to-severe AD and moderate-to-severe SD.

### Emotional Comparison Between AD and SD

Analysis of covariance (ANCOVA) was computed to explore differences between NC, AD and SD groups (see Table 3). Adjusting for years of education, analysis of covariance revealed significant group differences on AES and TEQ score. Compared to NC, patients with AD and SD got lower scores in AES and TEQ. In TEQ, mild AD performed better than mild SD (*p* = 0.030), and moderate-to-severe AD performed better than moderate-to-severe SD (*p* = 0.038). But in AES, the scores were not significantly different between AD and SD with the same severity. In addition, NC, AD, and SD got the similar scores in SAS and GDS.

**Table 3:** AS, GDS, AES, and TEQ performance of the groups.

**Index**	**NC (*n* = 39)**	**AD (*****n*** **= 83)**		**SD (*****n*** **= 43)**		***F* (*P*)**
		**Mild (*n* = 48)**	**Moderate-to-severe (*n* = 35)**	**Mild (*n* = 20)**	**Moderate-to-severe (*n* = 23)**	
SAS	35.75 (2.274)	38.82 (2.242)	38.80 (2.529)	33.48 (2.764)	35.38 (3.080)	0.210 (0.932)
		38.81 (2.386)		34.50 (2.922)		0.326 (0.723)
GDS	5.72 (1.040)	7.39 (1.093)	6.99 (1.127)	5.66 (1.281)	6.73 (1.399)	1.114 (0.353)
		7.22 (1.112)		6.23 (1.338)		1.723 (0.183)
AES	34.87 (3.127)^b^^,^^c^^,^^d^^,^^e^^,^^f^^,^^g^	25.30 (3.104)^a^^,^^c^^,^^e^	19.13 (3.039)^a^^,^^b^	18.087 (3.725)^a^	13.08 (3.765)^a^^,^^b^	4.813 (0.001)
		22.70 (3.071)^a^		15.41 (3.744)^a^		4.545 (0.013)
TEQ	43.66 (2.321)^b^^,^^c^^,^^d^^,^^e^^,^^f^^,^^g^	36.69(2.356)^a^^,^^d^^,^^e^	33.14(2.322)^a^^,^^e^	29.76(2.729)^a^^,^^b^	27.19(2.737)^a^^,^^b^^,^^c^	9.374 (0.000)
		35.19(2.338)^a^^,^^g^		28.39(2.734)^a^^,^^f^		16.845 (0.000)

*These variables compared by ANCOVA with post-hoc pairwise LSD tests*.

*The scores were presented as mean (standard deviation)*.

a *p < 0.05 compared to NC group*.

b *p < 0.05 compared to mild AD group*.

c *p < 0.05 compared to moderate to severe AD group*.

d *p < 0.05 compared to mild SD group*.

e *p < 0.05 compared to moderate-to-severe SD group*.

f *p < 0.05 compared to AD group*.

g *p < 0.05 compared to SD group*.

*SAS, Self-rating anxiety scale; GDS, Geriatric depression scale; AES, Apathy Evaluation Scale; TEQ, The toronto empathy questionnaire*.

### Correlations Between Emotional Scales and Other Cognitive Measures

Table 4 presents the correlation coefficients R-value between the four scales and other cognitive measures in dementia patients using correlation analysis. Both AES and TEQ correlated with MMSE, TEQ also correlated with delayed recall of complex figure test (CFT-DR), and correlation between GDS and delayed recall of auditory verbal learning test (AVLT-DR) was significant. There was no significant correlation between other emotional scales and cognitive measures.

**Table 4:** Correlations between emotional scales and other cognitive measures in dementia patients.

	**MMSE**	**MES**	**BNT**	**AFT**	**SDMT**	**reading**	**AVLT-DR**	**CFT-copy**	**CFT-DR**
SAS	0.115	0.069	0.011	−0.113	−0.118	0.045	0.182	−0.043	0.162
GDS	0.149	0.021	−0.011	−0.008	0.116	0.002	0.293^**^	0.095	0.119
AES	0.237^**^	0.186	0.061	0.222	0.131	−0.001	0.022	−0.151	−0.134
TEQ	0.282^**^	0.047	0.223	0.159	−0.072	0.218	−0.081	−0.206	0.316^*^

*R-value was presented in this table*.

* *Correlation is significant at the 0.05 level (two-tailed)*.

** *Correlation is significant at the 0.01 level (two-tailed)*.

## Discussion

This study is the first one to simultaneously assess apathy, empathy, depression and anxiety in patients with SD and AD. The results revealed both similarities and differences with past studies in which emotion was investigated in dementia patients. For example, similar to studies of apathy and empathy (7, 8, 16–18), the current study showed AD and SD patients got lower scores than healthy adults in AES and TEQ. Unlike those previous studies (3–5, 11–13), however, anxiety and depressive scores between dementia patients and healthy adults had no significant difference.

Empathy can be broadly defined as the ability to understand what others feel (cognitive empathy) and feel what others feel (emotional empathy). Patients with dementia have primarily cognitive impairments and also emotional deficits that lead to behavioral dysregulation (32), impairments in empathy will be evident in them. In order to compare it between AD and SD patients, it is informative to have two groups of patients who suffer similar cognitive deficits. In our study, the severity of cognitive impairment was close between mild AD and mild SD, as well as between moderate-to-severe AD and moderate-to-severe SD. Moreover, from our study, we found that in TEQ, mild AD performed better than mild SD (*p* = 0.030), and moderate-to-severe AD performed better than moderate-to-severe SD (*p* = 0.038). Therefore, we propose that emotional deficits were different between AD and SD, or a specific deficit may exist in SD patients. The exact mechanism is unclear and should be the topic of future studies. One possibility is that the cognitive and affective empathy may be mediated in different domains. At a neuroanatomical level, a broad network of structures has been implicated for empathy. Lesion studies have indicated that ventromedial frontal lesion result in deficits in cognitive empathy, yet deficits in emotional empathy most prominently arise from disturbances in the medial frontal cortex (such as the ventromedial prefrontal cortex and anterior cingulate gyrus and the anterior insula (33, 34). Besides, A meta-analysis in FTD showed that emotional empathy was also associated with amygdale and right anterior temporal lobe as well as corresponding neural networks (35). Some studies proposed that emotional empathy may arise from disease affecting precentral gyrus (36), orbitofrontal cortex (37), inferior parietal lobule, brainstem, and thalamus (38). Future studies should test the hypothesis.

An unexpected finding of our study was that anxiety and depressive scores between dementia patients and healthy adults had no significant differences. Interestingly, both SAS and GDS score did not correlate with almost all of the cognitive measures we did in our study. Maybe there were other factors that would increase the odds of anxiety and depression in dementia patients. For example, Hynninen et al. found that anxiety was not associated with cognitive test performance, but with depression, higher caregiver stress, and more dementia-related impairment (39). In addition, some researchers suggested that there were several areas where depression and dementia may overlap. Depression in a cognitively healthy older person may indicate early dementia, and depression may be a risk factor for dementia (40). Common biological and psychosocial risk factors for depression may exist among the cognitively intact as well as cognitively impaired older populations. For example, the interaction between an individual person and his environment is thought to play a role in the expression of depression and other behavioral and psychological symptoms (41). Therefore, an important issue regarding anxiety and depression in dementia is whether they should be considered as a separate clinical entity or as part of a broader syndrome.

One of the biggest strengths of the study is its employment of scales rating. However, this is also the study's biggest weakness: scale rating is highly subjective, solely based on individuals' reaction or opinion. For example in SAS, to the same degree of fatigue, someone choose “1,” but others choose “2.” This limitation, however, becomes less serious when more participants are tested. Thus, more participants should be recruited in future studies. Moreover, AD was diagnosed as probable AD according to the NINCDS-ADRDA/NINCDS-AIREN criteria, SD was diagnosed on the basis of clinical manifestation, and there were no distinctive biomarkers such as amyloid β (Aβ) or position-emission tomography (PET), so error could not be avoided.

## Data Availability Statement

The raw data supporting the conclusions of this article will be made available by the authors, without undue reservation.

## Ethics Statement

The studies involving human participants were reviewed and approved by the ethics committee of Shanghai Huashan Hospital Fudan University. The patients/participants provided their written informed consent to participate in this study.

## Author Contributions

PW, ZH, and QG designed the study and participated in the diagnoses of the diseases. JL and QG performed the data analysis. PW, QZ, and YZ participated in the evaluation of scales and interpretation of data. PW wrote the paper with input from all authors. All authors discussed the results and contributed to the final manuscript, revised the manuscript content, and approved the final version of the manuscript.

## Conflict of Interest

The authors declare that the research was conducted in the absence of any commercial or financial relationships that could be construed as a potential conflict of interest.
